# Recurrent Pseudomembranous Colitis in an Ovarian Cancer Patient Undergoing Carboplatin Chemotherapy

**DOI:** 10.1155/2016/7540302

**Published:** 2016-03-08

**Authors:** Valerie A. Allen, Kelly J. Manahan, John P. Geisler

**Affiliations:** Division of Gynecologic Oncology, Cancer Treatment Centers of America, Newnan, GA 30265, USA

## Abstract

*Background*. Diarrhea is a common problem in ovarian cancer patients undergoing chemotherapy and* Clostridium difficile* infection has been identified as a cause. The proper diagnosis and treatment of diarrhea are critical to patient care, especially to prevent the serious complications from a severe* Clostridium difficile* infection (CDI).* Case*. We present a heavily pretreated ovarian cancer patient who developed recurrent pseudomembranous colitis while receiving carboplatin chemotherapy. Despite treatment with oral metronidazole for fourteen days, the patient's diarrhea relapsed and colonoscopy revealed extensive pseudomembranous colitis. The infection eventually resolved with the combination of oral vancomycin and metronidazole.* Conclusions*. Diarrhea is a common problem in patients undergoing chemotherapy for ovarian cancer. Management requires obtaining the proper diagnosis.* Clostridium difficile* associated pseudomembranous colitis must be part of the differential diagnosis. Treatment must be sufficient to prevent relapses of the* Clostridium difficile* infection to prevent serious consequences in an already vulnerable patient population.

## 1. Introduction

*Clostridium difficile* infection (CDI) was recognized in 1978 as the etiology of antibiotic associated diarrhea and subsequent pseudomembranous colitis [1, 2]. In modern practice, clinical suspicion for CDI remains high when a patient develops diarrhea in close temporal relation to a course of antibiotics. However, in addition to antibiotics, chemotherapy has been confirmed as an independent risk factor for development of CDI and recently chemotherapy has become an established independent risk factor for healthcare associated* Clostridium difficile* colonization [3, 4].

The purpose of this paper is to describe a case of recurrent* Clostridium difficile* pseudomembranous colitis associated with single agent carboplatin chemotherapy in a woman with ovarian cancer.

## 2. Case Report

A 64-year-old woman with a long history of ovarian cancer presented with recurrent diarrhea after a fifth cycle of carboplatin. She had recently completed a fourteen-day course of oral metronidazole for* Clostridium difficile* colitis which was diagnosed after her fourth cycle of carboplatin chemotherapy.

The patient was originally diagnosed with Stage IIIc grade 3 papillary serous carcinoma of the ovary. She was treated with a primary suboptimal debulking by a surgical oncologist and subsequently had received six cycles of combination carboplatin (AUC 5) and paclitaxel (175 mg/m^2^). The ovarian carcinoma recurred within the first 12 months of completing this first-line therapy. She underwent a secondary suboptimal debulking by the same surgeon and was placed on daily ×5 topotecan followed by multiple other chemotherapy regimens including gemcitabine, capecitabine, weekly paclitaxel, and monthly liposomal doxorubicin. All of these chemotherapies were given in less than 12 months.

After going through all of the above regimens, she was referred to the gynecologic oncology service. After long discussion, knowing that her platinum-free interval was just over 20 months, she underwent a tertiary debulking with complete cytoreduction, including diaphragm resection, liver wedge resection, partial gastrectomy, infragastric omentectomy, lymph node dissection, splenectomy, and ablation of disease. Molecular profiling of the tumor revealed lack of potential benefit for all of the above named chemotherapies except carboplatin.

**Figure 1:**
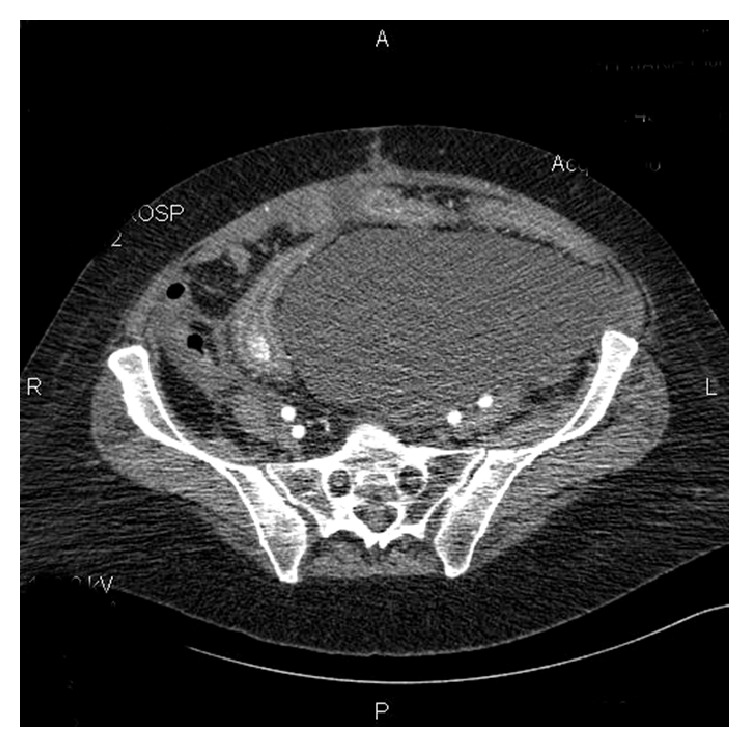
Ascites which had resolved from cancer recurred with* Clostridium difficile* infection.

She was started on carboplatin AUC 5 q 21 days. After her fourth cycle of carboplatin, despite a normalized CA 125, the patient presented with new onset ascites and diarrhea and was diagnosed with* Clostridium difficile* colitis. She was started on a 14-day course of oral metronidazole. She had resolution of her symptoms (ascites and diarrhea) and went on to get a fifth cycle of carboplatin with a 1-week delay. Two and a half weeks out from her fifth cycle of intravenous carboplatin she was admitted to the hospital with severe dehydration and diarrhea. She was mildly anemic (9.5 g/dL) and was not neutropenic (ANC 2.1 thousand/mm^3^). She was rehydrated with normal saline and restarted on oral metronidazole 500 mg by mouth every eight hours while awaiting the* Clostridium difficile* toxin assays. A computed tomography scan revealed severe colitis with ascites and new pleural effusion (see Figure 1).

**Figure 2:**
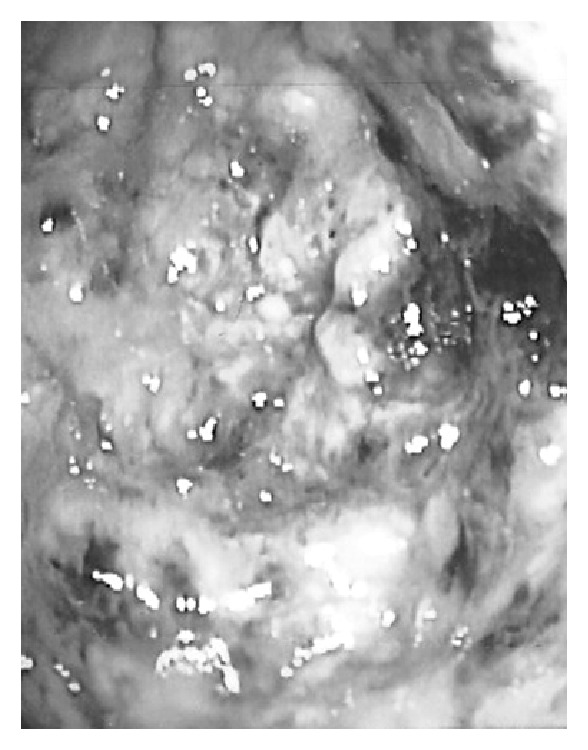
Highly inflamed colon (from colonoscopy) with pseudomembranes present.

A gastroenterology consult was ordered, and she underwent a colonoscopy which revealed severe pseudomembranous colitis consistent with* Clostridium difficile* colitis (Figure 2). She was started on oral vancomycin and maintained on the oral metronidazole. The colitis resolved and she went on to have a complete response from the carboplatin. She was maintained on once daily vancomycin during her sixth cycle of chemotherapy and did not suffer a relapse of the colitis. She went on to have a secondary progression-free interval/platinum-free interval of 24 months before requiring further therapy.

## 3. Discussion

In addition to chemotherapy, risk factors associated with healthcare associated* Clostridium difficile* colonization include previous hospitalization, use of proton-pump inhibitors or H2 blockers, and use of antibiotics against toxin B [5]. Patients who require chemotherapy generally have a history of hospital stays and thus have an increased risk to exposure of the bacteria. The increased risk of colonization by the bacteria predisposes the patient to CDI. Several chemotherapeutic agents have been implicated in postchemotherapy CDI including cisplatin, paclitaxel, carboplatin, and doxorubicin amongst many others [3, 4, 6, 7]. To the best of our knowledge, this case report represents the first incidence of CDI associated with carboplatin therapy alone, although others have developed theories that the risk of CDI may increase as a patient undergoes subsequent cycles of chemotherapy because the chemotherapy prevents the reestablishment of flora in the gut [4].

While the association of chemotherapeutic agents with CDI has been well established, the incidence of postchemotherapy recurrent CDI has not been well studied. Recurrences of CDI have been noted to occur with chemotherapy but not with a study of corresponding factors [8]. Anand and Glatt found a 43% incidence of recurrence in the seven cases they reviewed but noted no correlation of factors for these cases. In general, relapses of CDI are not entirely uncommon; 10–20% of patients treated for CDI will develop a relapse once treatment is discontinued [3]. The mechanism allowing recurrence is unclear but is postulated to be due to unsuccessful eradication of* Clostridium difficile* from the colon or nosocomial reinfection.

Standard diagnosis is by clinical presentation followed by toxins A and B being found in the stool. The role of computed tomography is not fully known but is currently being better defined [9]. Two independent risk factors from CT imaging were identified to predict the development of severe* Clostridium difficile* infection: pleural effusion and moderate-severe increased bowel wall thickness (>11 mm) [9].

In regard to CDI in relation to chemotherapeutic agents, Emoto et al. found that 6.1% (total of 2 patients) of their study patients developed* Clostridium difficile* colitis after cisplatin therapy for ovarian carcinoma, with one patient developing a relapse upon administration of a subsequent dose of cisplatin [10]. Both patients were subsequently treated with carboplatin and did not have further episodes of CDI. Our patient was treated with carboplatin and developed CDI. The patient was kept on the carboplatin after the development of CDI because of the great tumor response which had occurred and she was close to completing chemotherapy. If CDI had occurred earlier in the course of treatment, changing to cisplatin would have been considered although the value of changing chemotherapy to avoid CDI is unknown. With CDI becoming more common, it is important for all gynecologic oncologists to be aware of the clinical diagnosis and treatment.
